# Tourniquet use in arthroscopic anterior cruciate ligament reconstruction: a systematic review and meta-analysis of randomised controlled trials

**DOI:** 10.1186/s12891-017-1722-y

**Published:** 2017-08-22

**Authors:** Liang-Tseng Kuo, Pei-An Yu, Chi-Lung Chen, Wei-Hsiu Hsu, Ching-Chi Chi

**Affiliations:** 1 Department of Orthopedic Surgery, Division of Sports Medicine, Chang Gung Memorial Hospital, 6, Sec West, Chia-Pu Rd, Puzih, Chiayi, 61363 Taiwan; 2 Centre for Evidence-Based Medicine, Chang Gung Memorial Hospital, Chiayi, Taiwan; 3 Chang Gung University of Science and Technology, Chiayi, Taiwan; 4 College of Medicine, Chang Gung University, Taoyuan, Taiwan; 5 Department of Dermatology, Chang Gung Memorial Hospital, Linkou, 5, Fuxing St, Guishan Dist, Taoyuan, 33305 Taiwan

**Keywords:** Knee, Anterior cruciate ligament, Tourniquet, Arthroscopy, Systematic review, Meta-analysis

## Abstract

**Background:**

To assess the effects of tourniquet use in arthroscopic anterior cruciate ligament (ACL) reconstruction surgery.

**Methods:**

We conducted a systematic review and meta-analysis of randomised controlled trials (RCTs) that compared surgical outcomes following tourniquet use against non-tourniquet use during ACL reconstruction surgery. We searched the Cochrane Central Register of Controlled Trials, MEDLINE, and EMBASE for relevant RCTs. We used the Cochrane Collaboration’s tool to assess the risk of bias of included RCTs, and performed a random-effects meta-analysis in calculating the pooled risk estimates. The primary outcomes was postoperative pain measured by visual analogue scale, verbal rating scale, or required morphine dose. The secondary outcomes were blood loss in drainage, operative time, muscle strength, and calf and thigh girth.

**Results:**

We included 5 RCTs with 226 participants (116 in the tourniquet group and 110 in the non-tourniquet group). Postoperative pain and morphine doses were not significantly different between the two groups. Compared to the non-tourniquet group, the tourniquet group had a significantly increased blood loss in the drain (mean difference: 94.40 ml; 95% CI 3.65–185.14; *P* = 0.04). No significant differences in the operative time and muscle strength were found between the two groups. Tourniquet use was associated with a greater decrease in thigh girth but not in calf girth.

**Conclusions:**

The current evidence shows that compared to tourniquet use, ACL reconstruction surgery without tourniquet does not appear to have any major disadvantages and does not prolong operation time. There might be less drain blood loss associated with tourniquet use, though drains are no longer routinely used in ACL reconstruction surgery.

## Background

Whether a tourniquet should be used in arthroscopic anterior cruciate ligament (ACL) reconstruction surgery is unclear. The advantages of tourniquet use include less intra-articular blood loss, improved visualization, and hence potential shortening of operative time [1–5]. However, there are reports of adverse effects of tourniquet use. The disadvantages include increased postoperative pain, neuropathies, muscle weakness, and atrophy [6–8]. Rare complications like rhabdomyolysis and a high incidence of thromboembolic events have also been reported [9–11].

There are studies in favour of and against the use of tourniquet. Tsarouhas et al. [4] reported that tourniquet use did not affect postoperative pain and return to light work and jogging. Hoogeslag et al. [12] proposed that tourniquet use improved visibility in arthroscopic knee surgery. The meta-analyses and reviews of Smith and Hing [13] claimed that tourniquet use during arthroscopic ACL reconstruction improved surgical visualization but were contradicted by Zhang et al. [11], who found that it did not reduce blood loss or improve surgical visualisation. Wu et al. [5] published a systematic review on this issue, which reported no significant benefit in operative time using a tourniquet. However, due to heterogeneity over outcomes and limited data provided with a small number of participants (mainly based on two trials), only one outcome could be noted by meta-analysis, thus the value of tourniquet use remains largely unclear.

A recent randomised controlled trial (RCT) on tourniquet use has been published in 2014 [14]. Adding this new trial to systematic review make participant number larger and more synthesis of outcomes possible. To clarify the pros and cons of tourniquet use during ACL reconstruction, we performed a systematic review and meta-analysis of RCTs that evaluated the effects of tourniquet use on surgical outcomes including postoperative pain, muscle strength, blood loss, and operative time as well as thigh and calf girth. Our hypothesis was that the use of tourniquet would provide little benefits in ACL reconstruction surgery.

## Methods

### Data source and search strategy

We searched comprehensively for all RCTs that compared surgical outcomes following tourniquet use against non-tourniquet use during ACL reconstruction surgery. We searched the Cochrane Central Register of Controlled Trials, MEDLINE, and EMBASE for relevant RCTs from inception to February 20, 2017. The search terms were “(tourniquet OR tourniquets OR tourniquets [Mesh Terms]) AND (anterior cruciate ligament [MeSH Terms] OR (anterior AND cruciate AND ligament) OR anterior cruciate ligament)” without restrictions for language or gender. We also searched the U.S. National Institutes of Health trials register (https://clinicaltrials.gov). In addition, we contacted specialists in this field for any ongoing trial or relevant unpublished data on this topic.

Trials were included if they met the following criteria: (1) designed as a RCT; (2) compared surgical outcomes after using and not using a tourniquet during ACL reconstruction surgery. There were no restrictions on the graft type. Two authors (author A and author B) independently checked the citations identified from the searches against the inclusion criteria. The reference lists of included trials were also checked to identify relevant RCTs.

### Data extraction and quality assessment

Two authors (LTK and PAY) independently extracted the following data from the included trials using a standardized data extraction form: first author, year of publication, study design (patient selection and concealment), sample size, participant characteristics (e.g. age, sex, etc.), use of tourniquet (tourniquet pressure and time), and outcome data (subjective outcome: pain score; objective outcomes: operative time (min), blood loss through intra-articular drain (ml), morphine dose (mg), thigh girth (cm), calf girth (cm), and isokinetic quadriceps and hamstring strength). A third author (CCC) arbitrated when the two authors disagreed.

The same two authors independently used the Cochrane Collaboration’s tool to assess the risk of bias of included RCTs [15, 16]. A third author resolved differences of opinion. We assessed the following seven domains related to biased estimates of intervention effects: random sequence generation, allocation concealment, blinding of patient and personnel, blinding of outcome assessment, incomplete outcome data, selective reporting, and other biases [16]. For each domain, high risk of bias, low risk of bias, or unclear risk of bias was judged according to the quality of each RCT [15].

The primary outcome was the postoperative pain level on day one, which was judged using a patient-reported visual analogue scale (VAS), a verbal rating scale (VRS), or the required morphine dose. Secondary outcomes included operative time, blood loss in the drain, isokinetic quadriceps strength, and thigh and calf girth.

### Statistical analysis

Quantitative analysis was undertaken for pain intensity represented with postoperative morphine consumption dose within 24 h after surgery, operative time, blood loss, and Data were reported as mean difference (MD) or standardized mean difference (SMD) with a 95% CI for continuous data. The χ^2^ and I^2^ statistics were used to examine statistical heterogeneity: significance was set at *P* < 0.10. I^2^ values of 0–24.9%, 25–49.9%, 50–74%, and 75–100% were considered none, low, moderate, and high heterogeneity, respectively [17, 18]. We also estimated the between-study variance using tau-square (*τ* ^2^) statistic [16]. We used a random-effects model meta-analysis for all outcomes because we expected clinical heterogeneity across the included RCTs [19]. When analysing continuous data, if the standard deviation was not reported, we estimated the mean and variance from the reported median, range, and sample size [20]. When the standard deviation and range were not available, the variance was estimated from the *P* value in the *t* test. When only graphs were available without raw data for analysis, a software was used to extract the details [21]. A forest plot was applied for summary of results. The Review Manager 5.3 (The Nordic Cochrane Centre, The Cochrane Collaboration, 2014) was used for meta-analysis.

## Results

**Fig. 1:**
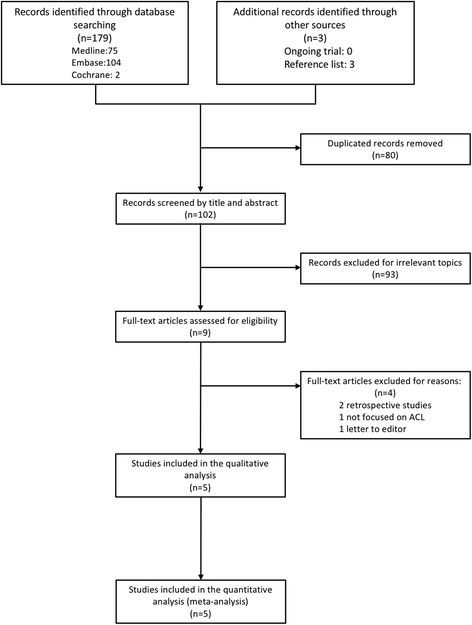
PRISMA flow diagram of the study

The PRISMA study flow diagram is shown in Fig. 1 [22]. We found 179 published RCTs after searching the MEDLINE, EMBASE, and CENTRAL databases. Three additional records were identified from the bibliography of included RCTs. After we had removed 80 duplicates and excluded 97 studies because they had irrelevant topics or a non-randomised design, we included 5 RCTs for this meta-analysis: Arciero et al. [1], Hooper et al. [3], Nakayama et al. [23], Nicholas et al. [24], and Reda et al. [14]. No ongoing trials were identified after consulting specialists and searching the trial register.

### Study characteristics

The included RCTs were published between 1996 and 2015 (Table 1). The sample sizes ranged from 29 to 58, with a total of 226 participants (116 in the tourniquet group and 110 in the non-tourniquet group). All the included studies reported how they used tourniquets. The tourniquet pressure ranged from 269 to 350 mmHg and the mean tourniquet time ranged from 69 to 86 min. For the non-tourniquet groups, diluted epinephrine was added to the irrigation solution in Nakayama et al. [23], Nicholas et al. [24], and Reda et al. [14]. ACL reconstruction was achieved with bone-patella-tendon-bone (BPTB) autografts in Arciero et al. [1] and Nicholas et al. [24]. Hamstring autografts were used in the remaining three. The types of anaesthesia used were different across trials and included: general (GA), epidural (EA), spinal anaesthesia (SA) (Table 1).

**Table 1:** Characteristics of included studies

First Author	Place/Year	Type of		Sample size	Gender ratio (M/F)		Mean age^d^		Epinephrine use in NT group	Tourniquet Setting		Follow-up time^f^
		Autograft	Anaesthesia	n (T/NT)	T	NT	T	NT		Pressure^d^	Duration^e^	
Arciero [1]	Canada/1996	BPTB	NR	40 (20/20)	13/7	17/3	24.8	26.7	NR	269	87 (64–105)	12
Hooper [3]	Canada/1999	Hamstring	GA	29 (14/15)	5/9	10/9	35.3	35.7	NR	300	NR	NR
Nakayama [23]	Japan/2013	Hamstring	GA^a^	51 (28/23)	11/17	16/7	26.0	22.0	1:3,000,000	300	NR	3
Nicholas [24]	USA/2001	BPTB	GA/EA^b^	48 (25/23)	13/12	16/7	33.0	32.0	1:100,000	300	85 ± 7.0	6
Reda [14]	Egypt/2015	Hamstring	SA	58 (29/29)	25/4	17/3	26.0	22.0	1:250,000	269	87 (64–105)	6

T tourniquet, NT non-tourniquet, M male, F female, BPTB bone-patella-tendon-bone, GA general anaesthesia, EA epidural anaesthesia, SA spinal anaesthesia, NR not reported

^a^An additional femoral nerve block using 20 ml of 0.25% bupivacaine was done for all participants

^b^GA = 38; EA = 16, ^c^Mean age in years, ^d^Pressure in mmHg, ^e^Duration in minutes, ^f^Follow-up in months

**Fig. 2:**
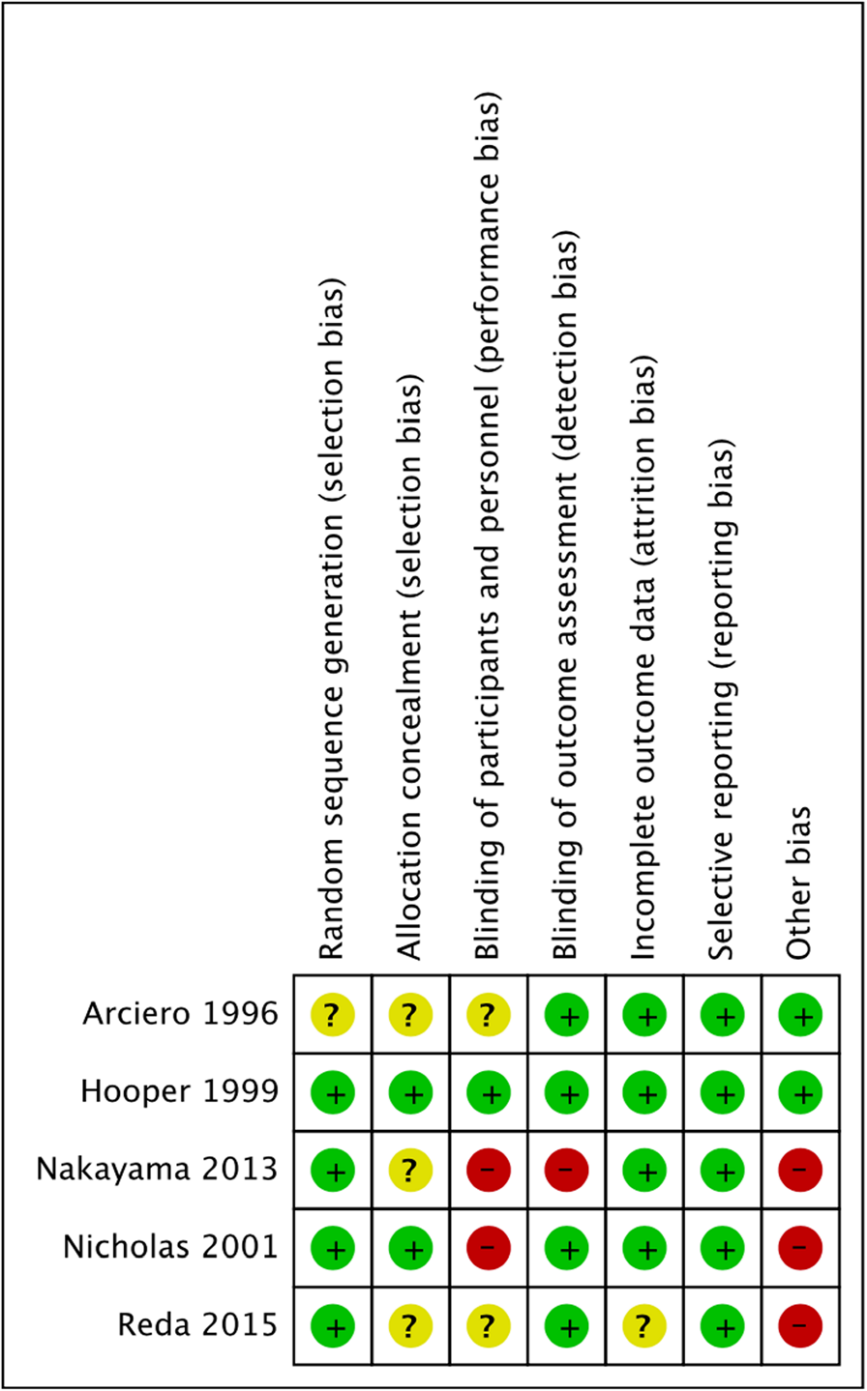
Risk of bias summary: authors’ judgments about each risk of bias item for each included study. The “−” sign means low risk of bias, the “+” sign means high risk of bias, and the “?” sign means unclear risk of bias

Double blinding was used only in Hooper et al. [3]. Blinding of participants and researchers (performance bias) in all trials was generally unclear or at a high risk, except in Hooper et al. [3]. Blinding of outcome assessment (detection bias) was generally at a low risk, except in Nakayama et al. [23]. All the included trials were at an unclear or a high risk of bias when appraised using the Cochrane Collaboration’s tool for assessing the risk of bias in RCTs [17, 18] (Fig. 2).

## Postoperative pain

### Pain score

**Fig. 3:**
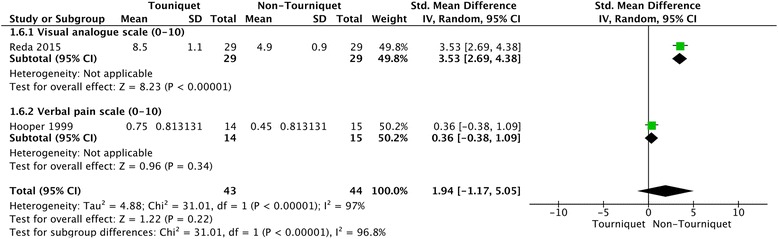
Forest plot of patient-reported pain score for tourniquet group versus non-tourniquet group. There was no significant difference in patient-reported pain score 6 h after surgery between the tourniquet and non-tourniquet groups

**Fig. 4:**
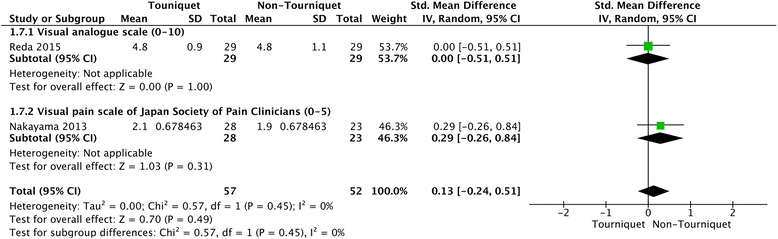
Forest plot of patient-reported pain score for tourniquet group versus non-tourniquet group. There was no significant difference in patient-reported pain score 22 h after surgery between the tourniquet and non-tourniquet groups

Only three studies with 138 patients reported postoperative patient-reported pain score [3, 14, 23]. Hooper et al. provided data on the VRS for pain within 6 h after surgery [3], and Reda et al. reported pain VAS (from 0 to 10) at 4, 10, 16 and 22 h following surgery [14], while Nakayama et al. reported pain VAS of the Japan Society of Pain Clinicians (from 0 to 5) 1 day following surgery [23]. The random-effects meta-analysis showed no significant differences between the two group in the patient-reported pain score at either 6 h (SMD = 1.94, 95% CI = −1.17 to 5.05, *P* = 0.22) or 22 h after surgery (SMD = 0.13, 95% CI = −−0.24 to 0.51, *P* = 0.49) (Figs. 3 and 4). The power for this outcome was 0.14.

### Required postoperative morphine dose

**Fig. 5:**
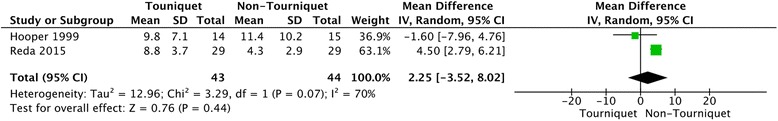
Forest plot of postoperative morphine consumption for tourniquet group versus non-tourniquet group. There was no significant difference in required postoperative morphine doses between the tourniquet and non-tourniquet groups

Hooper et al. [3] and Reda et al. [14] with a total 87 participants reported the required postoperative morphine doses within 24 h after surgery. A random-effects meta-analysis showed no significant difference in the mean dose between the two group (MD = 2.25 mg, 95% CI = −3.52 to 8.02, *P* = 0.44) (Fig. 5). The power for this outcome was 0.27. Statistical heterogeneity across the included RCTs was found (*τ* ^2^ = 12.96, *χ* ^*2*^ = 3.29, *P* = 0.07, I^2^ = 70%).

## Operative time

**Fig. 6:**
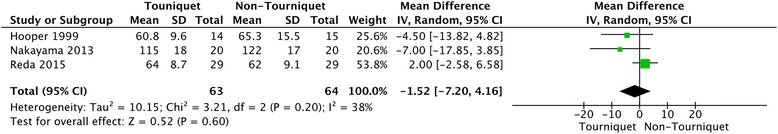
Forest plot of operation time for tourniquet group versus non-tourniquet group. There was no significant difference in postoperative operation time between the tourniquet and non-tourniquet groups

Only Hooper et al. [3], Nakayama et al. [23], and Reda et al. [14] with a total of 138 participants reported operative time. The tourniquet group appeared to have a slightly shorter operative time, but a meta-analysis using the random-effects model found no significant differences between the two groups (MD = 1.52 min when using a tourniquet, 95% CI = −7.20 to 4.16, *P* = 0.60) (Fig. 6). The power for this outcome was 0.37.

There was no significant heterogeneity across the RCTs (*τ* ^2^ = 10.15, *χ* ^*2*^ = 3.21, *P* = 0.20, I^2^ = 38%).

## Blood loss in the drain

**Fig. 7:**
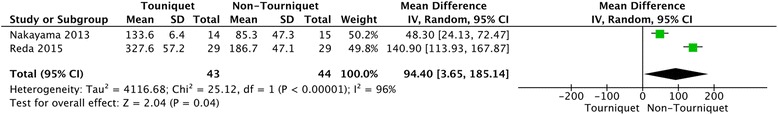
Forest plot of blood loss in surgical drain for tourniquet group versus non-tourniquet group. The tourniquet group had 94.4 ml more blood loss in the surgical drain than did the non-tourniquet group

Nakayama et al. [23] and Reda et al. [14] with a total of 109 participants reported blood loss in surgical drains during the first 24 h after surgery. There was significant heterogeneity across the trials (*τ* ^2^ = 4116.68, *χ* ^*2*^ = 25.12, *P* < 0.00001, I^2^ = 96%). Nakayama et al. [23] reported significantly more blood loss in the drain in the tourniquet group than in the non-tourniquet group (133.6 ± 6.4 ml vs. 85.3 ± 47.3 ml, *P* = 0.02). Reda et al. [14] also reported significantly more blood loss in the tourniquet group (327.6 ± 57.2 ml vs. 186.7 ± 47.1 ml, *P* = 0.001). Pooling the two trials together using a random-effects meta-analysis found more blood loss in the surgical drain in the tourniquet group than in the non-tourniquet group (MD = 94.40 ml, 95% CI = 3.65 to 185.14, *P* = 0.04) (Fig. 7).

## Muscle strength

Except for Hooper et al. [3], four included RCTs with a total of 197 participants reported postsurgical muscle strength. Nakayama et al. [23] and Reda et al. [14] reported that the isokinetic quadriceps and hamstring strengths were not significantly different between the two groups either before or after surgery. There were insufficient data available for meta-analysis.

**Fig. 8:**
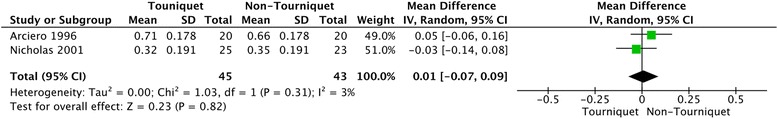
Forest plot of isokinetic quadriceps strength (60°/S) at 6 months after surgery for tourniquet group and non-tourniquet group. There was no significant difference in isokinetic quadriceps strength at 60°/S at 6 months after surgery between the tourniquet and non-tourniquet groups

Only Arciero et al. [1] and Nicholas et al. [24] reported detailed data on isokinetic muscle strength. A random-effects meta-analysis found no significant differences in isokinetic quadriceps strength measured at 60°/S at 6 months after surgery between the tourniquet and non-tourniquet groups (MD = 0.01 more than for a normal limb site compared with non-tourniquet patients, 95% CI = −0.07–0.09, *P* = 0.82) (Fig. 8). The power for this outcome was 0.47. There was no significant heterogeneity across the trials (*τ* ^*2*^ = 0.00, *χ* ^*2*^ = 1.03, *P* = 0.31, I^2^ = 3%).

## Thigh and calf girth

Three studies with a total of 157 participants evaluated postsurgical thigh and calf girth. Nakayama et al. [23] measured thigh girth at the level of the proximal pole of the patella, and found no significant side-to-side difference between the two groups. Nicholas et al. [24] and Reda et al. [14] both measured the thigh girth at the same level (1/3 the distance between the patella and the anterior superior iliac spine above the superior pole of the patella). Nicholas et al. [24] compared the thigh girth difference between healthy and non-healthy limb, and reported a greater decrease in thigh girth 3 weeks following surgery in the tourniquet group than that in the non-tourniquet group (mean decrease: 2.5 cm vs. 1.1 cm, *P* < 0.05). Reda et al. [14] also found that thigh girth in the tourniquet group decreased more than that in the non-tourniquet group 2 weeks after surgery (mean thigh girth 33.4 ± 1.9 cm vs. 35.6 ± 3 cm, respectively, *P* = 0.001). The available data were insufficient for meta-analysis.

Nicholas et al. [24] and Reda et al. [14] measured the calf girth at the same level (1/3 the distance from the lateral joint line to the lateral malleolus). The postsurgical decrease in calf girth was significantly different between the tourniquet and non-tourniquet groups in Reda et al. [14] (mean calf girth 30.9 ± 1.8 cm vs. 33.1 ± 3 cm, respectively, *P* = 0.001), but not significantly different in Nicholas et al. [24] (mean decrease: 1.1 cm versus 1.0 cm *P* = 0.78). There were insufficient data available for meta-analysis.

## Discussion

Our major finding was that tourniquet use during arthroscopic ACL reconstruction surgery significantly increased postoperative blood loss in the drain. There were no significant differences in postoperative pain, the required postoperative morphine dose, operative time, or quadriceps muscle strength at 6 months after surgery. That is, comparing to tourniquet use, ACL reconstruction surgery without tourniquet did not have major disadvantages and complications, and there might be less blood loss in the drain.

There are still debates on whether tourniquet use increases the intensity of postoperative pain and hence the amounts of analgesics administered. Some studies supported this notion [14, 25] but others did not [3, 23]. The findings of our study do not support the effect of tourniquet in worsening postoperative pain, no matter from the view of patient-reported pain score 6 h or 16 h after surgery or from the view of morphine doses within 24 h after surgery. Thus, tourniquet use appears not associated with increased postoperative pain.

The type of anaesthesia administered, especially regional anaesthesia (EA, SA and nerve block), did affect the levels of postoperative pain, but the effects on the tourniquet and non-tourniquet groups were comparable in most of the included RCTs [1, 3, 14, 23]. And the result of meta-analysis of morphine consumption was reported with mean difference between T and NT group, which also minimize the potential bias by type of anaesthesia. Only Nicholas et al. used a mixed type of anaesthesia, but no pain measurements were reported [24]. Two RCTs that reported morphine consumption used hamstring autografts but different types of anaesthesia (GA and SA) [3, 14]; however, it is sensible to combine these two studies because the effect of SA did not exceed 4–6 h after surgery.

Tourniquet use in arthroscopic surgery decreases intra-articular bleeding and increases surgical visualization, which potentially shortens operative time [26]. Reda et al. [14] and Hooper et al. [3] evaluated arthroscopic visualization during surgery. Only Hooper et al. [3] reported poorer arthroscopic visibility in the non-tourniquet group. However, satisfactory visualisation is possible by increasing the flow of irrigation fluid or adding epinephrine to the irrigation solution without having to inflate the tourniquet. The operative time did not significantly differ between the two groups. Also, in a recent systematic review that compared using and not using a tourniquet in arthroscopic ACL reconstruction, the operative times were not significantly different [5]. Our meta-analysis showed similar results, i.e. tourniquet use did not shorten the operative time.

We found that tourniquet use in arthroscopic ACL reconstruction surgery might increase blood loss in the drain by about 100 ml, which might cause discomfort during postoperative rehabilitation exercises. That is, a drain is necessary to prevent a large haemarthrosis and other potential complications when using a tourniquet in arthroscopic ACL reconstruction surgery. However, the association between tourniquet use and blood loss in the drain may not be applicable since the drain is not used in most ACL reconstruction surgery nowadays. Meanwhile, the three studies that reported blood loss in the drain had a major flaw [14, 23, 24]. The diluted epinephrine was added to irrigation solution in the non-tourniquet group but not in the tourniquet group. That is, tourniquet use was actually compared with not using a tourniquet plus using epinephrine solution. In other words, ACL reconstruction surgery without tourniquet, but instead using irrigation with epinephrine, is an alternative to tourniquet use in ACL reconstruction surgery.

Furthermore, compared to drainage volume, the haemoglobin level or calculated total blood loss is the preferable way to assess the intraoperative blood loss instead. However, except for Nakayama’s trial [23], no other trials employed the two outcomes in assessing intraoperative blood loss. Further study evaluating effect of tourniquet on intraoperative blood loss should report these two outcomes instead to make this issue clearer.

Muscle weakness and atrophy are other concerns related to tourniquet use. Some studies [14, 27–29] showed that using a tourniquet was associated with prolonged muscle weakness and delayed functional recovery. Although Reda et al. [14] reported a significant decrease in the thigh and calf girth, there were no significant differences in quadriceps muscle strength at 6 months after surgery between using and not using a tourniquet. Our study confirmed that tourniquet use was not associated with quadriceps muscle weakness.

None of the studies reported serious complications associated with tourniquet use, e.g. nerve palsy, vascular damage, and thromboembolic events. This might be attributable to the relatively small sample size in each RCT, all of which lacked adequate power to detect these rare complications (i.e. Type II error). The question of whether tourniquet use for arthroscopic ACL reconstruction is safe remains unanswered based on the current available evidence.

### Strengths and limitations

This meta-analysis has some strengths: (1) it is the most updated systematic review and meta-analysis of RCTs to explore the effects of tourniquet use in arthroscopic ACL reconstruction; (2) we comprehensively searched the three largest and most comprehensive databases for relevant RCTs; (3) all included RCTs were assessed using the Cochrane Collaboration’s tool for the risk of bias; (4) for all but one outcome included in the meta-analyses, no heterogeneity was observed between the two groups.

Our meta-analysis also has some limitations: First, our study only included one additional study than previous systematic review by Wu et al. [5]. However, our study provides more information via meta-analysis including patient-reported pain score, morphine dose, blood loss, and isokinetic quadriceps strength than previous systematic review. Second, the included studies in this review did not have sufficient data for all outcomes available for meta-analysis. The pain score level was one of the primary outcomes of interest. However due to variation of measures and lack of data in some studies, we could not perform a meta-analysis and could not compare the data between studies. Third, due to relatively low methodological quality, small sample size, and clinical heterogeneity of clinical setting and outcome assessment across included studies, it should be very cautious while applying these results to clinical practice. Larger-scale high-quality RCTs might be warranted to clarify this issue.

## Conclusions

The current evidence shows that compared to using tourniquet, ACL reconstruction surgery without tourniquet, but using irrigation with epinephrine instead, does not appear to have any major disadvantages and does not prolong operation time. There might also be less drain blood loss; though drains are no longer routinely used in ACL reconstruction surgery.

## Abbreviations

ACL: anterior cruciate ligament
EA: epidural anaesthesia
GA: general anaesthesia
MD: mean difference
RCT: randomised controlled trial
SA: spinal anaesthesia
VAS: visual analogue scale
VRS: verbal rating scale
